# Burden of Diabetic Retinopathy amongst People with Diabetes Attending Primary Care in Kerala: Nayanamritham Project

**DOI:** 10.3390/jcm10245903

**Published:** 2021-12-16

**Authors:** Sobha Sivaprasad, Vasudeva Iyer Sahasranamam, Simon George, Rajeev Sadanandan, Bipin Gopal, Lakshmi Premnazir, Dolores Conroy, Jyotsna Srinath, Radha Ramakrishnan, Sundaramuthil Murukaiah Vijayanand, Raphael Wittenberg, Gopalakrishnan Netuveli

**Affiliations:** 1 NIHR Biomedical Research Centre, Moorfields Eye Hospital NHS Foundation Trust, London EC1V 2PD, UK; 2 Regional Institute of Ophthalmology, Thiruvananthapuram 695035, India; drsahasranamam@gmail.com (V.I.S.); simongeorge1986@yahoo.com (S.G.); 3 Health Systems Transformation Platform, New Delhi 110070, India; rsadanandan@gmail.com; 4 Directorate of Health Services, Thiruvananthapuram 695035, India; bipingopal@yahoo.com (B.G.); lakshmipremnazir08@gmail.com (L.P.); smvijayanand@yahoo.com (S.M.V.); 5 UCL Institute of Ophthalmology, London EC1V 9EL, UK; d.conroy@ucl.ac.uk (D.C.); r.ramakrishnan@ucl.ac.uk (R.R.); 6 Institute for Connected Communities, University of East London, London E16 2RD, UK; j.srinath@uel.ac.uk (J.S.); g.netuveli@uel.ac.uk (G.N.); 7 Centre for Health Service Economics and Organisation, Nuffield Department of Primary Care Health Sciences, University of Oxford, Oxford OX2 6GG, UK; raphael.wittenberg@phc.ox.ac.uk

**Keywords:** diabetic retinopathy, Kerala, India, diabetes, screening, socio-economic status, risk factors

## Abstract

Background: The burden of diabetic retinopathy (DR) in people attending the public health sector in India is unclear. Thirty percent of the population in India is reliant on public healthcare. This study aimed to estimate the prevalence of DR and its risk factors in people with diabetes in the non-communicable disease registers who were attending the family health centres (FHCs) in the Thiruvananthapuram district in Kerala. Methods: This cross-sectional study was conducted over 12 months in 2019 within the framework of a pilot district-wide teleophthalmology DR screening programme. The age- and gender-adjusted prevalence of any DR and sight-threatening DR (STDR) in the whole sample, considering socio-demography, lifestyle and known clinical risk groups, are reported. Results: A total of 4527 out of 5307 (85.3%) screened in the FHCs had gradable retinal images in at least one eye. The age and gender standardised prevalence for any DR was 17.4% (95% CI 15.1, 19.7), and STDR was 3.3% (95% CI 2.1, 4.5). Ages 41–70 years, males, longer diabetes duration, hyperglycaemia and hypertension, insulin users and lower socio-economic status were associated with both DR outcomes. Conclusions: The burden of DR and its risk factors in this study highlights the need to implement DR screening programs within primary care to reduce health inequality.

## 1. Introduction

Kerala has one of highest prevalence of diabetes amongst all states in India. Approximately 10% of adults in Kerala are estimated to have type 2 diabetes (T2DM) [1,2]. The state has undergone significant economic and health transitions over the last 30 years [3]. However, the triad of increasing wealth, improved lifestyle and reduced physical activity has contributed to the rising prevalence of T2DM and its complications [2].

Diabetic retinopathy (DR) is a common complication of diabetes and an avoidable cause of blindness [4]. Currently, approximately 4.5% of blindness in India is due to sight-threatening DR (STDR) [4,5]. Early identification and treatment of STDR reduces the risk of blindness. As a largely asymptomatic condition, people with diabetes have to be screened regularly for DR [6]. Systematic screening for DR in India is in its infancy. 

Most affluent people can access private healthcare in India and are therefore more likely to be screened for DR. In comparison, people who are socially disadvantaged, economically challenged and systemically marginalised rely mainly on the public health system [7]. In the absence of systematic DR screening in the public health system, the prevalence of DR in people who attend the public health system is not known. As primary care infrastructure is underdeveloped in India, data from primary care are scanty. 

The government of Kerala has significantly revamped the primary care in the public health system by introducing family health centres (FHCs) with electronic health records (eHealth) [8,9]. For people with diabetes, a comprehensive diabetes care plan was initiated about five years ago, and it includes screening for all complications of diabetes except DR [10]. Therefore, implementing a DR screening programme within the primary care not only provides a complete preventive medicine plan for people with diabetes but also contributes to achieving some of the Sustainable Development Goals (SDG) [11]. The SDG 3 is to achieve health and well-being for all people, and SDG 7 is to reduce inequalities by 2030.

The burden of DR and its risk factors in Kerala will also provide information on resource allocation for systematic DR screening in the primary care in the public health system.

In this cross-sectional study, we report the prevalence of any DR, STDR and referable retinopathy and associated risk factors in newly screened people with diabetes within the FHCs in the Thiruvananthapuram district in Kerala.

## 2. Methods

### 2.1. Study Design and Setting

The Government of Kerala collaborated with Moorfields Eye Hospital in the ORNATE India project funded by the Global Challenge Research Fund and United Kingdom Research and Innovation to set up the Nayanamritham project [12]. This pilot teleophthalmology DR care pathway was implemented for people utilising the public health system. Screening for DR was offered for people with diabetes attending the FHCs in the Thiruvananthapuram district and treatment for STDR delivered in secondary care hospitals [13]. This is a cross-sectional study of all individuals with diabetes registered in the non-communicable diseases (NCD) register who participated in the teleophthalmology DR screening program of Nayanamritham project in the 16 FHCS of Trivandrum District, Kerala over 12 months in 2019. Each FHC independently maintained a NCD register. 

### 2.2. Participants

Individuals with diabetes aged 30 years or above were identified from the NCD register and invited to participate in the DR screening program when they attended the FHCs for their diabetes care.

### 2.3. Data Acquisition

A study-specific questionnaire was administered to each patient by data entry operators at each FHC. Individual data included demographics, education, lifestyle (smoking, alcohol and physical activity), family history, blood pressure, body mass index (BMI) and waist circumference (WC). The participants also answered questions on their perception of their quality of life and vision. Self-reported history of macrovascular and microvascular complications, coronary heart disease, stroke, diabetic neuropathy and diabetic kidney disease was also collected. On the day of screening, blood pressure and either random blood glucose (RBG) or fasting blood glucose (FBG) were measured, and urine samples were tested for presence of albumin.

### 2.4. Diabetic Retinopathy Screening Protocol

When people with diabetes attended the FHCs for their regular diabetes care, both eyes were dilated with 1% tropicamide before retinal photography. Retinal images were captured by existing non-ophthalmic-trained primary care staff using indigenous smartphone-enabled retinal cameras (Remidio Fundus on Phone (FOP; Remidio Innovative Solutions Pvt. Ltd., Bengaluru, India) fixed on a frame and used as a tabletop device. This camera is compliant with European Conformity (CE marked) and the Health Insurance Portability and Accountability Act (HIPAA) and has been previously validated and used in several countries [14,15]. The FHC staff (nurses and doctors) were trained on the study protocol, the DR screening and referral pathway, mydriasis, capturing good quality retinal images and DR grades. Certificates of completion of training were issued by the University of East London, United Kingdom.

The retinal images were transferred through a newly established picture archiving and communication system (PACS) to the Regional Institute of Ophthalmology (RIO), the tertiary ophthalmic centre located in Thiruvananthapuram. For each eye, two images were taken and for most individuals images were taken for both eyes. The retinal images were graded at RIO by two certified graders, supervised by retinal specialists. The retinal graders were certified as accredited graders following completion of an online DR screening course, offered by the Gloucestershire NHS Foundation Trust [16].

The retinal photographs were first graded for quality of images based on the proportion of retina visible in the image available for grading. Four categories were used to describe gradeability: 100% gradable, 75% gradable, 50% gradable and less than 50% gradable, the latter of which was defined as ungradable. 

For gradable images, the severity of DR was graded according to the International DR severity grading as no DR, mild non-proliferative diabetic retinopathy (NPDR), moderate NPDR, severe NPDR and proliferative diabetic retinopathy (PDR) [17]. PDR was further classified into stable treated and active PDR requiring laser photocoagulation and advanced diabetic eye disease (vitreous haemorrhage, tractional retinal detachment, rhegmatogenous retinal detachment, iris or angle neovascularisation, neovascular glaucoma and blindness due to DR. Diabetic macular oedema (DMO) was graded as per the definitions of clinically significant DMO as absent or present [17]. People with ungradable retinal images were also referred to secondary care for further evaluation.

### 2.5. Outcomes

The prevalence of the following in the study sample adjusted for age and gender was analysed.

1. Any DR was defined as presence of any grade of NPDR, PDR or DMO in at least one eye of an individual.
2. Referable retinopathy included severe NPDR, PDR or DMO in any or both eyes of an individual.
3. DMO in any or both eyes of an individual.
4. STDR was defined as presence of PDR and/or DMO.

Secondary analysis included prevalence of these outcomes in each FHC as well as in sub-populations defined by socio-demographic, lifestyle and clinical factors.

### 2.6. Sample Size

Our sample consisted of all those who attended DR screening at the 16 FHCs during the study period. Using an estimated DR prevalence figure of 10%, we estimated, using Cochran’s sample size formula, that we would need a sample of 3458 individuals to estimate that level of prevalence with 1% error. To offset any loss to the sample due to ungradable images and incompleteness of data collected by newly trained data operators, we planned to recruit at least 5000 people with diabetes.

### 2.7. Statistical Analysis

Since the sample was drawn from NCD registers maintained independently in each FHC, there was the potential for clustering effects in the sample. We accounted for any clustering effect in the analyses by setting up the data as complex survey data with each FHC as the primary sampling unit.

### 2.8. Estimation of Prevalence of DR

The prevalence was calculated adjusted by age and gender. As the standard errors from direct standardization would not account for clustering, we used predicted marginal probabilities for the purpose of prevalence calculation (Stata command margins after logistic regression with age and gender and command contrast for testing statistical significance between categories). We report the prevalence for the whole sample screened for sub-populations defined by known risk factors and for each of the FHCs. We also fitted a multivariable logistic regression to test the strength of association of the risk factors to the outcomes. 

To identify if the FHCs cluster was based on the prevalence of any DR and STDR we did a k-means cluster analysis of the prevalence figures from 15 FHCs. This sample size might be adequate based on Formann’s rule of thumb, 2*^p^*, where *p* is the number of variables (two in our case) [18].

### 2.9. Risk Factor Analysis

#### 2.9.1. Socio-Demographic Variables

Age was categorised into the following groups: 31–40 years, 41 to 50 years, 51 to 60 years, 61 to 70 years or more than 70 years. Other variables considered were gender, education (none, primary, secondary or graduate and higher) and occupation (not working, housewife, retired, unskilled worker, skilled worker, professional and self-employed). We created a binary variable for self-reported income above and below INR 600, the sample median income. 

#### 2.9.2. Diabetes Variables

These included parental history of diabetes (none, either one of the parents or both parents having diabetes), duration of diabetes (less than 4 years, 4 to 9 years or more than 9 years since first diagnosis of diabetes), whether insulin was used in the treatment or not, having at least one complication of diabetes pre DR screening and a categorical variable indicating uncontrolled diabetes based on fasting blood glucose (FBG ≥ 7 mmol/L) or random blood glucose (RBG ≥ 11.1 mmol/L). 

#### 2.9.3. Behavioural Risk Factors and Covariates

Behaviours relating to smoking, physical activity and diet were combined to create a healthy lifestyle score. Smoking was scored as 0 for smokers, 0.5 for ex-smokers and 1 for non-smokers. Physical activity was scored as 1 for those participating in activities of moderate or severe intensity and 0 otherwise. Dietary habits were scored based on a checklist of five unhealthy dietary habits, namely intake of salty snacks, fried snacks, fruits less than once a day, vegetables less than once a day and meat and poultry more than twice a day. The diet score was 0 if three or more items were checked, 0.5 if two items were checked and 1 if one or no item was checked. Healthy lifestyle score was a summative score based on these three scores and further dichotomised at the median. 

#### 2.9.4. Clinical Risk Factors and Covariates

Obesity status was categorised according to the Asian criteria, with a body mass index (BMI) 18.5–22.9 as normal, overweight as 23–24.9 and obese as ≥25. Waist circumference was dichotomised according to the above WHO-recommended cut off points (for women: ≥88 cm and for men: ≥102 cm) [19]. Hypertension status of each subject was classified by systolic blood pressure (SBP) according to the 2017 Guidelines for High Blood Pressure in Adults from the American College of Cardiology and American Heart Association (Hypertension stage 1 SBP 130–139 mmHg, Hypertension stage 2 SBP > 139 mmHg) [20]. We also noted the presence of other complications of diabetes such as neuropathy and chronic kidney disease. If the patient reported that they were told they had DR previously, it was also noted. Analyses were performed using STATA 15.1 (StataCorp LLC, College Station, TX, USA).

## 3. Results 

The study complied with the Declaration of Helsinki and was approved by The Indian Council of Medical Research (ICMR)/Health Ministry Screening Committee (HMSC/(2018-0551) dated 13/03/2019. Written informed consent was obtained from each participant.

**Figure 1:**
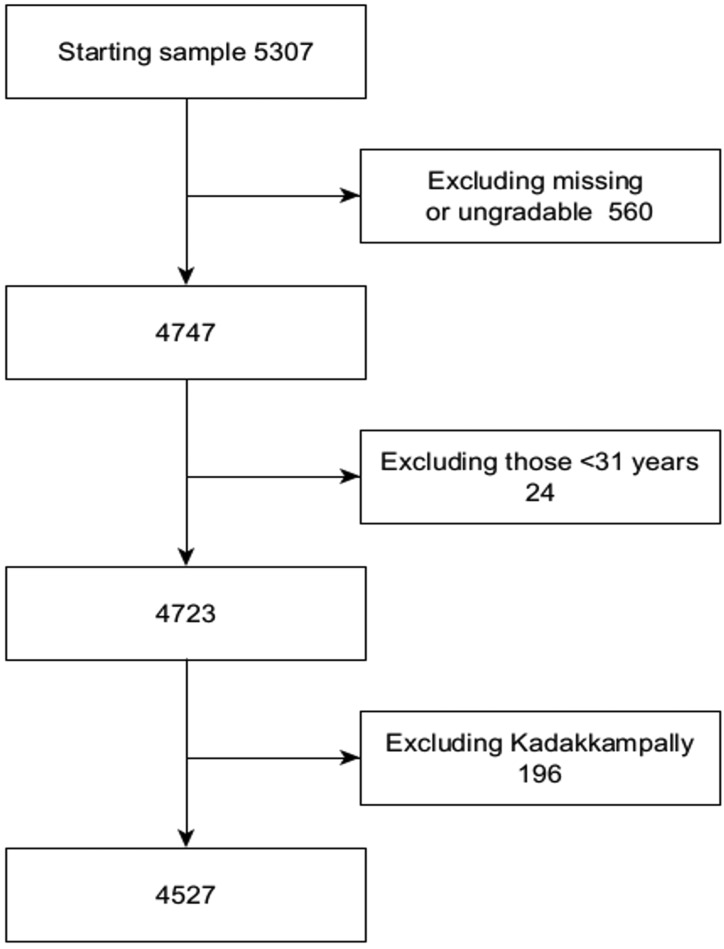
Flow diagram showing derivation of the study sample size.

A total of 5307 individuals with diabetes in the NCD registers at the 16 FHCs were screened. All those who did not meet the eligibility criteria or had missing primary outcome data were excluded. One FHC (Kadakkampally) was excluded due to outlying results which could not be validated by re-examination (Table 1). The final sample size was 4527 (85.3%). The flow diagram (Figure 1) shows the derivation of the study sample size.

Table 2 compares the demographic, socio-economic and clinical characteristics of all those screened (*N* = 5307) versus the working sample (*N* = 4527, 85.3%), which excluded those with ungradable or unreliably graded images. The distributions of the variables in the working sample closely matched those of the whole screened population. Seventy percent of the participants were aged between 51 and 70 years and two-thirds of them were females. Ninety-five percent of the participants had only school-level education, although Kerala had a high proportion of enrolment in higher education. Compared to the general population, the sample in this study reported considerably lower income, which is not surprising considering more than half of the sample represented non-workers or housewives. Nearly 75% of the sample were overweight or obese and 40% had a waist circumference indicative of central obesity. The blood pressure was normal in only forty percent and the diabetes was uncontrolled in 60%. Neuropathy was the commonest complication after hypertension, while less than 1% had been told about DR previously. More than a quarter of the patients had insulin as a part of their treatment. Seventy-five percent of the participants had had diabetes for a duration of five years or more.

### 3.1. Prevalence of DR 

The prevalence of any DR was 17.4% (95% CI 15.1%, 19.7%) and STDR had a prevalence of 3.3% (95%CI 2.1%, 4.5%) (Table 3). The prevalence of DMO was 2.3% (95%CI 1.3%, 3.3%) suggesting that nearly two-thirds of STDR was contributed by DMO. Adding severe NPDR to STDR, the prevalence of DR referable to secondary or tertiary care was 8.3% (95%CI 6.3%, 10.1%), nearly half the cases with any DR.

The age distribution of any DR was ‘n’ shaped with lower prevalence for the extreme age ranges of 31–40 years and 70+ years. (Table 3) The lowest prevalence was seen in the category aged 70 years or above. Both STDR and referable DR followed the same distribution. In contrast, DMO showed a declining trend with age (Chi-square for trend df = 1, 11.245, *p* < 0.001). The prevalence of any DR was higher in males (t = 3.87, *p* = 0.002). Other outcomes did not show this distinction. Age- and gender-standardised prevalence of DR in each FHC is shown in Table A1.

### 3.2. Age- and Gender-Standardised Prevalence of DR in Socio-Demographic Groups 

The prevalence of DR outcome was different according to socio-demographic groups in some instances (Table 4). The prevalence for any DR was 13.6% (95%CI 10.0%, 17.1%) in the highest education group compared to those who had no education (18.8%, 95%CI 11.5%, 26.0%; *p* = 0.097). When occupation is considered, those in professional group had lower prevalence of DR (9.8%, 95% CI 2.7%, 16.9%) compared to those not working (20%, 95%CI 16.5%, 23.4%; *p* = 0.032). The prevalence of referable DR was 10.2% (95% CI 8.1%, 12.3%) in the lower income group compared to the higher income group (6.6%, 95%CI (5.0%, 8.2%; *p* ≤ 0.001) and also among housewives (7.7%, 95%CI 6.1%, 9.4%) compared to those who were not working (12% 95%CI 8.1%, 15.9%, *p* = 0.042).

There were no differences in the prevalence of any of the DR outcomes according to the categories of healthy lifestyle score and waist circumference (Table 5). However, in the case of BMI, compared to those with normal BMI (22.2%, 95%CI 19.1%, 25.3%), both overweight (17.5% 95%CI 14.3%, 20.7%; *p* = 0.005) and obese (13.2% 95%CI 10.7%, 15.7%; *p* < 0.001) showed a significantly lower prevalence of any DR. A similar pattern was seen also for the prevalence of referable DR (overweight *p* = 0.003; obese *p* < 0.001). 

When we consider clinical association (Table 5), significant differences were seen in those with stage 2 hypertension (systolic blood pressure ≥ 140 mmHg) for any DR (*p* = 0.039) and neuropathy for referable DR (*p* = 0.002).

Subpopulations based on diabetic-related factors except parental history showed the greatest prevalence of DR (Table 6). The prevalence of any DR was significantly higher in those with a parental history of diabetes compared to those who did not (19.3% vs. 16.4%, *p* = 0.022). 

**Figure 2:**
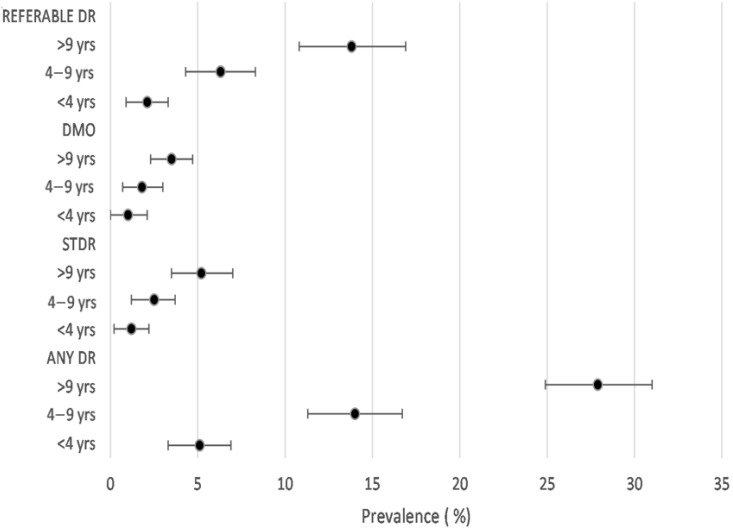
Age- and sex-standardised prevalence of DR outcomes according to duration of diabetes.

In case of the duration of diabetes all outcomes showed trends towards an increase in the prevalence of DR with increasing duration of diabetes (*p* < 0.001 (visualised in Figure 2). The prevalence of all DR outcomes was significantly higher in those on insulin treatment and those who reported a previous diagnosis of DR. In addition, patients whose diabetes was not controlled had a significantly higher prevalence of DR outcomes in comparison to those whose diabetes was controlled (*p* < 0.001 in all comparisons).

### 3.3. DR Associations 

There were only a few significant associations between DR and DMO with socio-demographic variables (Table 7). Of interest, higher education (OR 0.81, 95% CI 0.66, 0.996) and income (OR 0.66 95% CI 0.52, 0.85) have protective effects on STDR.

Being on insulin is an indicator of high risk of DR (mild to moderate DR: OR 2.05 95%CI 1.67, 2.52, severe DR: OR 2.48 95% CI 2.12, 2.92, and STDR: OR 2.08 95% CI 1.64, 2.65). Hyperglycaemia, especially indicated by random blood glucose, was significant. Among the diabetic complications, neuropathy (OR 1.43 95% CI 1.13, 1.80) and diabetic kidney disease (OR 4.38 95% CI 2.98, 6.46) increased the risk of STDR. Unlike macrovascular complications, both high BMI and waist circumference appeared to be protective. The duration of diabetes was significantly associated with DR and DMO with risks increasing linearly with duration. High systolic blood pressure was significantly associated DR but not with DMO or STDR. There were fewer significant associations between the risk factors and DMO in comparison with DR. These factors were hyperglycaemia indicated by random blood glucose, diabetic kidney disease and duration of diabetes.

## 4. Discussion

This study reports the prevalence of DR, STDR, DMO and referable DR in a sample of people with diabetes registered in the NCD register across 15 out of 16 FHCs in the Thiruvananthapuram district in Kerala, where a mydriatic DR teleophthalmology service was set up as a pilot project to evaluate the burden of DR. Primary care in the public health sector is freely accessible but is predominantly accessed by the lower socio-economic strata who cannot afford private healthcare, the major provider of healthcare in India. The study, revealing the prevalence data for the poor, points to the need to ensure access to treatment for persons with DR without catastrophic out-of-pocket payment.

We report that the age- and gender-adjusted prevalence of DR is 17.4%, similar to prevalence data reported from population-based studies in India (range 12–20%) [21,22,23,24,25]. These figures, although providing less data than those reported in developed countries, are alarming. They show that 3 in 100 people with diabetes who attend the FHCs are at risk of visual impairment due to DR. Furthermore, 8% required referral to secondary ophthalmic care for further assessment even though only 10% of the acquired retinal images were ungradable. These results highlight that the burden of eye pathologies other than DR is also high, emphasising the urgent need to establish DR screening and treatment services in the public health system to prevent visual impairment in people with diabetes. 

The subpopulations that were more at risk of DR included people aged between 40–70 years, particularly males. This population is the working age group, and visual impairment in this group is likely to have a significant impact on the individual, their family and society. As the public health system is largely accessed by the lower socio-economic strata and our results show that DR, STDR and DMO are more prevalent in low-income groups, it further underscores the need for systematic screening for DR to be implemented in the public health to prevent health inequity. 

There are reports of the urban–rural divide in terms of prevalence of DR in India with rural residents having a lower prevalence of DR [25]. The 16 FHCs covered both urban and rural areas in Thiruvananthapuram district and due to the urbanisation of the whole district, such dissociations are challenging to decipher from this study sample. However, we ensured that the clustering effect of sampling from 16 FHCs were accounted for in our analysis. 

Although, the national standards on the proportion of ungradable images in mydriatic DR screening in the more established programme in high- income countries are set at less than 5% [26], and this teleophthalmology service is set up in low- and middle-income countries (LMIC), where most reports using non-mydriatic retinal imaging show an ungradable rate of less than 30% [27,28]. Cataracts remain a major challenge, and LMIC and DR screening programmes should be used as an opportunity to also identify cataract and other non-DR causes as these conditions are more prevalent than DR [29]. 

In our study, 8% of those screened required referral for DR, the greater proportion of which were referred due to severe NPDR rather than STDR, which only accounted for 3.5% of referrals. However, when we consider the total number of participants that required referral, about 20% required referral with 10% being referred due to ungradable images, highlighting that DR screening does detect eye conditions other than STDR that require attention and resources for management [30]. We do not expect all 20% will require treatment, as some of the ungradable images may be due to the technical failure of not obtaining gradable images [30]. Therefore, these figures may be an over-representation of referable DR. 

The sample is representative of the diabetes population at risk of DR because about 75% had lived more than 4 years with known diabetes, 60% had uncontrolled diabetes and about a third had systolic BP ≥ 140 mmHg and were already on insulin treatment [31]. About half of the sample had a family history of diabetes, and a third was already known to have another diabetes complication. Therefore, the study sample depicts the people in the low-socio-economic strata in Kerala with several high-risk characteristics of DR, STDR and DMO, highlighting the importance of DR screening in the FHCs. Although men are at higher risk of STDR, they are underrepresented (33%) in this sample. This observation may indicate that males utilise the primary care services less than women. 

Another point of consideration is that only 42 (0.8%) of the study participants with DR were aware that they had DR before the study, highlighting the importance of improving public awareness of DR-related blindness and the need for publicly funded systematic DR screening. 

The strengths of the study are that it is the first study on DR conducted within the public health system in Kerala on a large population sample. Quality assurance was ensured in the study by repeated training, data monitoring and quality checks. Furthermore, the study is representative of the population attending the FHCs, allowing the extrapolation of the requirements for a publicly funded DR screening programme in the FHCs [32]. 

The limitations of the study are that although consecutive individuals were meant to be invited to participate in the DR screening programme, the workload of the staff at the FHCs often did not permit such robustness, and hence this is best described as a convenience sample within each FHCs. Therefore, selection bias may have been introduced. These limitations also highlight the difficulties faced in implementing a DR screening programme in the context of resource constraints in a LMIC. However, during the 12- month study period, we were able to screen approximately 10% of the individuals with diabetes registered with the FHCs. 

## 5. Conclusions

This study highlights the burden of STDR and its risk factors in the public sector that mainly provides care to people in the low-socio-economic strata. Resources should be allocated to scale up and sustain a state-wide diabetic eye disease screening and treatment programme in Kerala. 

## Figures and Tables

**Table 1:** The representativeness of the sample at each FHC.

Family Health Centre (FHC)	Population Served by FHC	Numbers (%) or People with Diabetes	Screened Population *N* (%) 5307
Amachal	42,240	2768 (6.5%)	140 (5%)
Aruvikkara	37,555	4017 (10.6%)	412 (10.2%)
Balaramapuram	37,185	4202 (11.3%)	538 (12.8%)
Chemmaruthi	37,188	4122 (11.1%)	323 (7.8%)
Kadakampalli *	37,233	596 (1.6%)	306 (51.3%)
Karakulam	68,408	1864 (2.7%)	353 (18.9%)
Keezhattingal	29,522	842 (2.8%)	264 (31.3%)
Kilimanoor	24,894	1632 (6.5%)	390 (23.8%)
Kottukal	36,527	3280 (8.9%)	479 (14.6%)
Kuttichal	20,012	946 (4.7%)	92 (9.7%)
Pallichal	47,118	2841 (6%)	453 (15.9%)
Paraniyam	19,046	492 (2.5%)	199 (40.4%)
Poozhanad	19,253	2645 (13.7%)	299 (11.3%)
Thonnakkal	33,423	3468 (10.3%)	353 (10.1%)
Vamanapuram	28,865	1262 (4.3%)	310 (24.5%)
Vattiyoorkavu	56,830	3944 (6.9%)	396 (10%)

* Data excluded from study due to poor quality.

**Table 2:** Sample description.

Variables	All Screened (100%)	Working Sample (85.3%)
	*N* (%)	*N* (%)
Age categories (years)	5307 (100)	4527 (100)
<30	25 (0.5)	Excluded
31–40	177 (3.3)	142 (3.1)
41–50	877 (16.5)	763 (16.9)
51–60	1907 (35.9)	1691 (37.4)
61–70	1808 (34.1)	1547 (34.2)
>70	513 (9.7)	384 (8.5)
Gender	5307 (100)	4527 (100)
Female	3538 (66.7)	3023 (66.8)
Male	1769 (33.3)	1504 (33.2)
Education	5298 (100)	4521 (100)
None	476 (9.0)	401 (8.9)
Primary	2578 (48.7)	2159 (47.8)
Secondary	1943 (36.7)	1733 (38.3)
Graduate or higher	301 (5.7)	228 (5.0)
Income	5016 (100)	4275 (100)
<INR 600	2662 (53.1)	2250 (52.6)
>INR 600	2354 (46.9)	2025 (47.4)
Occupation	5306 (100)	4527 (100)
Not Working	772 (14.6)	616 (13.6)
Housewife	2549 (48.0)	2184 (48.2)
Retired	343 (6.5)	295 (6.5)
Unskilled worker	736 (13.9)	652 (14.4)
Skilled Worked	336 (6.3)	282 (6.2)
Professional	155 (2.9)	114 (2.5)
Self employed	415 (7.8)	384 (8.5)
Healthy lifestyle score (Median = 1.5)	5204 (100)	4450 (100)
Below median	2664 (51.2)	2333 (52.4)
Above median	2540 (48.8)	2117 (47.6)
BMI	5082 (100)	4337 (100)
Normal	1212 (23.9)	1042 (24.0)
Overweight	2450 (48.2)	2078 (47.9)
Obese	1420 (27.9)	1217 (28.1)
Waist circumference	5177(100)	4402 (100)
Below WHO cut-off	3124 (60.3)	2565 (58.3)
Above WHO cut-off	2053 (39.7)	1837 (41.7)
Systolic Blood Pressure	5223 (100)	4458 (100)
≤129 mmHg	1997 (38.2)	1764 (39.6)
130–139 mmHg	1307 (25.0)	1117 (25.1)
≥140 mmHg	1919 (36.7)	1577 (35.4)
Known history of Neuropathy	5307 (100)	4527 (100)
No	3739 (70.0)	3245 (71.7)
Yes	1568 (30.0)	1282 (28.3)
Known history of Diabetic kidney disease	5307 (100)	4527 (100)
No	5182 (97.6)	4419 (97.6)
Yes	125 (2.4)	108 (2.4)
Parental history of Diabetes	4462 (100)	3788 (100)
None	2312 (51.8)	1974 (52.1)
Either or both parents are diabetic	2150 (48.2)	1814 (47.9)
Duration of diabetes	5307 (100)	4527 (100)
<4 years	1322 (24.9)	1103 (24.4)
4 to 9 years	1775 (33.5)	1530 (33.8)
>9 years	2210 (41.6)	1894 (41.8)
Insulin used in treatment	5305 (100)	4525 (100)
No	3883 (73.2)	3303 (73.0)
Yes	1422 (26.8)	1222 (27.0)
FPG ≥ 7 mmol/L or RBG ≥ 11.1 mmol/L	4559 (100)	3934 (100)
No	1814 (39.8)	1540 (39.2)
Yes	2745 (60.2)	2394 (60.9)
Self-reported previous diagnosis of DR	5307 (100)	4527 (100)
No	5265 (99.2)	4497 (99.3)
Yes	42 (0.8)	30 (0.7)
Family Health Centre	5307 (100)	4527 (100)
Amachal	140 (2.6)	130 (2.9)
Aruvikkara	412 (7.8)	401 (8.9)
Balaramapuram	538 (10.1)	487 (10.8)
Chemmaruthi	323 (6.1)	269 (5.9)
Kadakampalli	306 (5.8)	Excluded
Karakulam	353 (6.7)	318 (7.0)
Keezhattingal	264 (5.0)	255 (5.6)
Kilimanoor	390 (7.4)	331 (7.3)
Kottukal	479 (9.0)	475 (10.5)
Kuttichal	92 (1.7)	73 (1.6)
Pallichal	453 (8.5)	394 (8.7)
Paraniyam	199 (3.8)	182 (4.0)
Poozhanad	299 (5.6)	263 (5.8)
Thonnakkal	353 (6.7)	312 (6.9)
Vamanapuram	310 (5.8)	255 (5.6)
Vattiyoorkavu	396 (7.5)	382 (8.4)

Abbreviations: FBG—fasting blood glucose; RBG—random blood glucose; BMI—body mass index; INR—Indian rupees; WHO—World Health Organization.

**Table 3:** Age- and gender-standardised prevalence of DR (*N* = 4527).

	Any DR	STDR	DMO	Referable DR
	(% (95%CI))	(% (95%CI))	(% (95%CI))	(% (95%CI))
Overall	17.4 (15.1, 19.7)	3.3 (2.1, 4.5)	2.3 (1.3, 3.3)	8.3 (6.5, 10.1)
Age group (years)				
31–40	15.6 (8.8, 22.4)	2.8 (0.2, 5.4)	2.8 (0.2, 5.4)	6.4 (2.1, 10.6)
41–50	19.6 (15.7, 23.4)	3.4 (1.7, 5.1)	2.6 (1.1, 4.1)	8.6 (5.8, 11.4)
51–60	18.4 (16, 20.8)	3.5 (2.0, 5.0)	2.3 (1.2, 3.4)	9 (7.0, 11.0)
61–70	17.4 (14.7, 20)	3.2 (2.0, 4.3)	2.1 (1.1, 3.1)	8.5 (6.7, 10.2)
>70	9.9 (5.7, 14.1)	2.8 (0.2, 5.4)	2.1 (0.0, 4.6)	5.1 (1.7, 8.5)
Gender				
Female	16.0 (13.4, 18.6)	3.2 (1.9, 4.5)	2.3 (1.2, 3.4)	7.8 (6.0, 9.7)
Male	20.4 (18, 22.8)	3.5 (2.3, 4.7)	2.3 (1.3, 3.2)	9.3 (6.9, 11.8)

Abbreviations: DR—diabetic retinopathy, STDR—sight-threatening retinopathy, DMO—diabetic macular oedema, Referrable DR—referrable diabetic retinopathy.

**Table 4:** Age- and gender-standardised prevalence of DR in socio-demographic groups (*N* = 4527).

	Any DR	STDR	DMO	Referable DR
	(% (95%CI))	(% (95%CI))	(% (95%CI))	(% (95%CI))
Education				
None	18.8 (11.5, 26.0)	2.8 (0.6, 5)	2.3 (0.3, 4.4)	9.6 (6.3, 13)
Primary	17.2 (14.4, 20.1)	3.4 (1.5, 5.3)	2.5 (0.8, 4.1)	8.8 (6.3, 11.2)
Secondary	17.8 (15.8, 19.8)	3.1 (2.3, 4.0)	2.1 (1.4, 2.8)	7.7 (5.8, 9.6)
Graduate or higher	13.6 (10.0, 17.1)	3.9 (0.8, 6.9)	2.1 (0.0, 4.4) ^ns^	7.1 (3.4, 10.8)
Income (Median = INR 600)				
Below median	18.3 (14.9, 21.8)	3.8 (1.8, 5.8)	2.7 (1.1, 4.3)	10.2 (8.1, 12.3)
Above median	16.4 (14.2, 18.7)	2.7 (1.9, 3.6)	2.0 (1.2, 2.7)	6.6 (5.0, 8.2)
Occupation				
Not working	20 (16.5, 23.4)	4.2 (1.9, 6.6)	2.2 (1.2, 3.1)	12 (8.1, 15.9)
Housewife	17.3 (14.0, 20.6)	3.5 (2.0, 4.9)	2.5 (1.2, 3.9)	7.7 (6.1, 9.4)
Retired	19.4 (13.9, 25)	3.7 (1.1, 6.4)	3.1 (0.9, 5.3)	9.1 (4.8, 13.4)
Unskilled worker	16.5 (12.8, 20.1)	3.5 (1.3, 5.6)	2.6 (0.7, 4.5)	8 (4.8, 11.2)
Skilled Worker	16.3 (11.3, 21.4)	2.9 (0.0, 6.2) ^ns^	1.6 (0.0, 3.9) ^ns^	7.3 (3.2, 11.4)
Professional	9.8 (2.7, 16.9)	1.6 (0.0, 4.0) ^ns^	0.8 (0.0, 2.5) ^ns^	3.2 (0.0, 7.5) ^ns^
Self employed	17.8 (15.3, 20.3)	1.2 (0.0, 2.9) ^ns^	1.2 (0.0, 2.9) ^ns^	8.4 (6.1, 10.7)

^ns^ not significant.

**Table 5:** Age- and gender-standardised prevalence of DR according to lifestyle factors (*N* = 4527).

	Any DR	STDR	DMO	Referable DR
	(% (95%CI))	(% (95%CI))	(% (95%CI))	(% (95%CI))
Healthy lifestyle score (Median = 1.5)				
Below median	17.3 (14.5, 20.2)	3.8 (1.9, 5.6)	2.5 (0.8, 4.3)	8.8 (6.0, 11.6)
Above median	17.4 (14.9, 20)	2.8 (1.4, 4.2)	2.1 (0.9, 3.2)	8.0 (6.5, 9.4)
BMI				
Normal	22.2 (19.1, 25.3)	4.0 (2.3, 5.7)	2.4 (0.7, 4.1)	11.1 (8.4, 13.8)
Overweight	17.5 (14.3, 20.7)	3.1 (1.7, 4.4)	2.1 (1.2, 3)	8.0 (6.0, 10.1)
Obese	13.2 (10.7, 15.7)	2.7 (1.2, 4.3)	2.4 (1.1, 3.7)	5.7 (4.1, 7.3)
Waist circumference				
Below WHO cut-off	18.7 (15.1, 22.3)	3.7 (1.8, 5.6)	2.7 (1.1, 4.2)	9.4 (6.5, 12.2)
Above WHO cut-off	15.3 (13.4, 17.2)	2.5 (1.6, 3.4)	1.7 (0.9, 2.4)	6.8 (5.0, 8.6)
Systolic Blood Pressure				
≤129 mmHg	15.5 (12.9, 18.0)	2.6 (1.1, 4.1)	1.9 (0.7, 3.1)	7.5 (5.8, 9.3)
130–139 mmHg	17.4 (14.8, 20.0)	3.5 (2.0, 5.0)	2.4 (1.1, 3.7)	8.2 (6.1, 10.3)
≥140 mmHg	20.0 (15.6, 24.5)	4.0 (2.3, 5.6)	2.7 (1.3, 4.0)	9.4 (6.9, 12.0)
History of Neuropathy				
No	16.4 (13.3, 19.5)	3.0 (1.5, 4.4)	2.3 (1.0, 3.5)	6.9 (5.2, 8.7)
Yes	20.1 (16.9, 23.4)	4.1 (2.5, 5.6)	2.4 (1.3, 3.4)	11.9 (9.0, 14.9)
History of diabetic kidney disease				
No	17.3 (15.0, 19.7)	3.3 (2.1, 4.5)	2.3 (1.3, 3.3)	8.2 (6.4, 10.1)
Yes	21.4 (11.8, 30.9)	3.7 (0, 10.0)	2.8 (1.4, 4.3)	12.1 (2.4, 21.8)

**Table 6:** Age- and sex-standardised prevalence of DR according to diabetes-related factors (*N* = 4527).

	Any DR	STDR	DMO	Referable DR
	(% (95%CI))	(% (95%CI))	(% (95%CI))	(% (95%CI))
Parental history of diabetes				
None	16.4 (13.6, 19.2)	3.1 (1.7, 4.4)	2.3 (1.2, 3.4)	8.1 (6.3, 10.0)
Either or both parents diabetic	19.3 (16.8, 21.8)	3.7 (2.1, 5.4)	2.5 (1.1, 3.9)	9.3 (7, 11.7)
Duration of diabetes				
<4 years	5.1 (3.3, 6.9)	1.2 (0.2, 2.2)	1.0 (0.0, 2.1)	2.1 (0.9, 3.3)
4 to 9 years	14 (11.3, 16.7)	2.5 (1.2, 3.7)	1.8 (0.7, 3)	6.3 (4.3, 8.3)
>9 years	27.9 (24.9, 31)	5.2 (3.5, 7)	3.5 (2.3, 4.7)	13.8 (10.8, 16.9)
Insulin used in treatment				
No	12.2 (10.4, 13.9)	2.5 (1.3, 3.6)	1.8 (0.7, 2.9)	5.6 (4.2, 7.0)
Yes	31.4 (27.3, 35.6)	5.6 (3.6, 7.5)	3.6 (2.2, 5.0)	15.5 (11.4, 19.5)
Hyperglycaemia				
FPG < 7 mmol/L or RBG < 11.1 mmol/L	13.6 (11.6, 15.7)	2.5 (0.9, 4.2)	1.9 (0.4, 3.4)	6.5 (4.6, 8.3)
FPG ≥ 7 mmol/L or RBG ≥ 11.1 mmol/L	20.1 (17.2, 22.9)	4.0 (2.5, 5.4)	2.8 (1.8, 3.9)	10.1 (8.2, 12.1)
Self-reported previous diagnosis of DR				
No	17.2 (14.9, 19.5)	3.2 (2.0, 4.4)	2.3 (1.3, 3.3)	8.1 (6.3, 9.9)
Yes	52.5 (40.8, 64.2)	16.7 (5.8, 27.6)	6.8 (1.6, 12.0)	36.3 (23.9, 48.7)

**Table 7:** Associations of DR with mutually adjusted socio-demographic and diabetes-related factors (*N* = 5307).

	Any DR (*N* = 2959)	STDR (*N* = 2959)	DMO (*N* = 2891)	Referable DR (*N* = 2959)
	OR (95%CI)	OR (95%CI)	OR (95%CI)	OR (95%CI)
Age group (years)				
<40	Reference	Reference	Reference	Reference
41–50	1.25 (0.72–2.16)	1.11 (0.31–3.9)	0.93 (0.29–2.97)	0.91 (0.44–1.89)
51–60	0.92 (0.49–1.74)	0.87 (0.21–3.56)	0.65 (0.15–2.79)	0.72 (0.32–1.62)
61–70	0.72 (0.39–1.34)	0.57 (0.14–2.27)	0.36 (0.10–1.23)	0.57 (0.26–1.23)
>70	0.39 (0.17–0.84)	0.91 (0.18–4.44)	0.74 (0.13–4.26)	0.36 (0.15–0.83)
Gender				
Female	Reference	Reference	Reference	Reference
Male	1.13 (0.88–1.46)	1.07 (0.53–2.18)	1.07 (0.57–2.04)	0.90 (0.66–1.27)
Education				
None	Reference	Reference	Reference	Reference
Primary	1.24 (0.76–2.02)	1.03 (0.37–2.84)	0.71 (0.25–2)	1.03 (0.62–1.70)
Secondary	1.09 (0.65–1.84)	0.82 (0.27–2.51)	0.55 (0.16–1.85)	0.84 (0.46–1.51)
Graduate or Higher	1.10 (0.64–1.91)	1.55 (0.39–6.07)	0.75 (0.15–3.72)	1.14 (0.55–2.34)
Income				
Below median	Reference	Reference	Reference	Reference
Above median	0.81 (0.63–1.06)	0.61 (0.37–0.98)	0.68 (0.41–1.14)	0.61 (0.46–0.82)
Occupation				
Not Working	Reference	Reference	Reference	Reference
Housewife	0.95 (0.61–1.47)	1.62 (0.52–4.98)	2.65 (0.75–9.29)	0.75 (0.45–1.24)
Retired	1.24 (0.90–1.72)	1.61 (0.58–4.44)	3.51 (0.799–15.41)	1.31 (0.80–2.13)
Unskilled Worker	0.97 (0.66–1.41)	1.47 (0.51–4.18)	2.30 (0.63–8.46)	0.76 (0.39–1.48)
Skilled Worker	0.88 (0.53–1.46)	0.99 (0.18–5.39)	1.08 (0.12–9.16)	0.73 (0.34–1.54)
Professional	0.50 (0.18–1.44)	0.51 (0.05–4.93)	1	0.43 (0.08–2.24)
Self-Employed	1.08 (0.71–1.62)	0.55 (0.09–3.10)	1.11 (0.14–8.99)	0.84 (0.43–1.65)
Lifestyle				
Healthy Lifestyle Score				
Below median	Reference	Reference	Reference	Reference
Above median	1.01 (0.76–1.34)	0.77 (0.32–1.84)	0.90 (0.29–2.79)	0.92 (0.58–1.46)
BMI				
Normal	Reference	Reference	Reference	Reference
Overweight	0.82 (0.60–1.13)	1.04 (0.73–1.49)	1.26 (0.82–1.94)	0.70 (0.55–0.88)
Obese	0.51 (0.38–0.69)	0.86 (0.46–1.63)	1.18 (0.62–2.27)	0.47 (0.33–0.67)
Waist circumference				
Normal	Reference	Reference	Reference	Reference
Above WHO cut-off	0.78 (0.62–1)	0.70 (0.39–1.24)	0.70 (0.35–1.39)	0.83 (0.60–1.15)
Comorbidities				
Neuropathy				
No	Reference	Reference	Reference	Reference
Yes	0.96 (0.69–1.32)	1.19 (0.67–2.12)	0.84 (0.44–1.61)	1.41 (1.07–1.85)
Chronic Kidney Disease				
No	Reference	Reference	Reference	Reference
Yes	0.92 (0.54–1.60)	0.53 (0.05–5.17)	0.87 (0.09–7.90)	0.75 (0.28–2.03)
Systolic Blood Pressure				
≤129 mmHg	Reference	Reference	Reference	Reference
130–139 mmHg	1.24 (0.97–1.59)	1.42 (0.71–2.81)	1.28 (0.58–3.13)	1.21 (0.86–1.71)
≥140 mmHg	1.37 (1.04–1.80)	1.68 (0.87–3.24)	1.50 (0.67–3.38)	1.43 (1.07–1.91)
Diabetes factors				
Positive family history				
No	Reference	Reference	Reference	Reference
Yes	1.17 (0.91–1.52)	1.27 (0.83–1.95)	1.02 (0.59–1.75)	1.25 (1.01–1.55)
Diabetes duration				
Less than 4 years	Reference	Reference	Reference	Reference
4 to 9 years	2.24 (1.52–3.29)	1.74 (0.86–3.53)	1.87 (0.94–3.73)	2.02 (1.09–3.74)
More than 9 years	4.44 (2.64–7.47)	2.58 (1.44–4.63)	2.22 (1.11–4.44)	3.93 (2.04–7.54)
Treatment including insulin				
No	Reference	Reference	Reference	Reference
Yes	2.54 (1.83–3.52)	2.09 (1.12–3.92)	1.68 (0.64–4.40)	2.71 (1.89–3.87)
Hyperglycaemia				
FPG <7 mmol/L) or RBG < 11.1 mmol/L)	Reference	Reference	Reference	Reference
FPG ≥ 7 mmol/L) or RBG ≥ 11.1 mmol/L)	1.38 (1.11–1.72)	1.07 (0.50–2.28)	1.10 (0.47–2.55)	1.26 (0.93–1.69)

Abbreviations: BMI—Body mass Index, DR—diabetic retinopathy, STDR—sight-threatening retinopathy, DMO—diabetic macular oedema, WHO—World Health Organisation, RBG—random blood glucose, FPG—fasting plasma glucose.

## Data Availability

The technical text statistical code and dataset will be made available on request after obtaining permission from the Government of Kerala.

## Patient and Public Involvement

Individuals of the public were involved in the conduct of the study, reporting and dissemination plans of our research.

## Appendix A

**Table A1:** Age- and gender-standardised prevalence of DR in each FHC.

	Sample	Any DR (% (95%CI))	STDR (% (95%CI))	DMO (% (95%CI))	Referable DR (% (95%CI))
Amachal	130	18.7 (18.3, 19.1)	3.0 (2.9, 3.2)	1.5 (1.4, 1.6)	6.8 (6.6, 7.0)
Aruvikkara	401	15.2 (15, 15.5)	4.2 (4.2, 4.3)	3 (2.9, 3)	9.8 (9.6, 9.9)
Balaramapuram	487	18.9 (18.6, 19.2)	2.7 (2.6, 2.8)	1.2 (1.2, 1.3)	6 (5.9, 6.2)
Chemmaruthi	269	18.5 (18.3, 18.8)	2.6 (2.6, 2.6)	2.6 (2.6, 2.7)	4.5 (4.4, 4.5)
Karakulam	318	19.5 (19.2, 19.7)	3.2 (3.1, 3.2)	1.6 (1.5, 1.6)	14.7 (14.4, 15)
Keezhattingal	255	19.5 (19.2, 19.9)	2.3 (2.2, 2.4)	1.6 (1.5, 1.7)	6.8 (6.6, 6.9)
Kilimanoor	331	13.7 (13.5, 13.9)	1.8 (1.8, 1.9)	1.5 (1.5, 1.6)	3.7 (3.6, 3.7)
Kottukal	475	17.2 (16.8, 17.5)	0.4 (0.4, 0.4)	0.4 (0.4, 0.4)	8.3 (8.2, 8.5)
Kuttichal	73	25.4 (25.1, 25.7)	2.7 (2.6, 2.8)	2.7 (2.6, 2.8)	12.1 (11.8, 12.3)
Pallichal	394	14.8 (14.5, 15.2)	4.1 (4.0, 4.2)	1.7 (1.7, 1.8)	8.5 (8.2, 8.7)
Paraniyam	182	17.5 (17.2, 17.7)	1.6 (1.6, 1.7)	1.1 (1.1, 1.1)	9.3 (9.2, 9.4)
Poozhanad	263	30.2 (29.7, 30.7)	9.7 (9.3, 10)	8.1 (7.8, 8.4)	15.2 (14.8, 15.6)
Thonnakkal	312	19.5 (19.2, 19.8)	5.4 (5.2, 5.6)	4.2 (4.0, 4.4)	10.4 (10.1, 10.6)
Vamanapuram	255	10.4 (10.2, 10.7)	1.6 (1.5, 1.6)	1.5 (1.4, 1.6)	6.2 (6.0, 6.4)
Vattiyoorkavu	382	12.6 (12.4, 12.7)	4.4 (4.3, 4.5)	3.1 (3.1, 3.2)	6.5 (6.4, 6.6)

Abbreviations: DR-Any diabetic retinopathy, STDR-sight-threatening diabetic retinopathy, DMO-diabetic macular oedema, Referable DR-referable diabetic retinopathy.
